# In Vivo Evaluation of a Novel Oriented Scaffold-BMSC Construct for Enhancing Full-Thickness Articular Cartilage Repair in a Rabbit Model

**DOI:** 10.1371/journal.pone.0145667

**Published:** 2015-12-22

**Authors:** Shuaijun Jia, Ting Zhang, Zhuo Xiong, Weimin Pan, Jian Liu, Wei Sun

**Affiliations:** 1 Department of Mechanical Engineering, Biomanufacturing Engineering Research Institute, Tsinghua University, Beijing, China; 2 Department of Orthopaedics, Shannxi Hospital of Armed Police Force, Xi'an, Shannxi, China; 3 Department of Human Movement Studies, Xi’an physical education university, Xi'an, Shannxi, China; 4 Institute of Orthopaedics, Xijing Hospital, The Fourth Military Medical University, Xi'an, Shannxi, China; 5 Department of Mechanical Engineering, Drexel University, Philadelphia, Pennsylvania, United States of America; University of California, UNITED STATES

## Abstract

Tissue engineering (TE) has been proven usefulness in cartilage defect repair. For effective cartilage repair, the structural orientation of the cartilage scaffold should mimic that of native articular cartilage, as this orientation is closely linked to cartilage mechanical functions. Using thermal-induced phase separation (TIPS) technology, we have fabricated an oriented cartilage extracellular matrix (ECM)-derived scaffold with a Young's modulus value 3 times higher than that of a random scaffold. In this study, we test the effectiveness of bone mesenchymal stem cell (BMSC)-scaffold constructs (cell-oriented and random) in repairing full-thickness articular cartilage defects in rabbits. While histological and immunohistochemical analyses revealed efficient cartilage regeneration and cartilaginous matrix secretion at 6 and 12 weeks after transplantation in both groups, the biochemical properties (levels of DNA, GAG, and collagen) and biomechanical values in the oriented scaffold group were higher than that in random group at early time points after implantation. While these differences were not evident at 24 weeks, the biochemical and biomechanical properties of the regenerated cartilage in the oriented scaffold-BMSC construct group were similar to that of native cartilage. These results demonstrate that an oriented scaffold, in combination with differentiated BMSCs can successfully repair full-thickness articular cartilage defects in rabbits, and produce cartilage enhanced biomechanical properties.

## Introduction

Articular cartilage, which forms the frictionless surface of diarthrodial joints, functions in load transmission in the joints [1]. Due to its avascular nature and low cell density, it shows limited capacity to regenerate or self-repair in adults [2]. Various surgical treatments, including microfracture, mosaicplasty, and autologous chondrocyte implantation (ACI) have been used clinically to relieve pain and repair cartilage defects [3]. However, these treatments are limited in efficacy, as they do not produce organized hyaline cartilage, but predominantly fibrous cartilage that lacks suitable mechanical properties [4].

Tissue engineering (TE), which involves combining isolated stem cells with degradable scaffolds and certain environmental factors, has the potential to be effective in restoring cartilage function [5, 6]. Recent advances in this area involve the development of sponge-like porous scaffolds derived from native biomaterials [7, 8]. However, the mechanical strength of these isotropic scaffolds is insufficient in supporting joint function during tissue regeneration, and the biomechanical properties of TE cartilage are consequently lower than that of native cartilage [9]. These deficiencies in construct structure and biomechanical function have restricted the clinical usefulness of TE cartilage [10], and the development of cartilage scaffolds with load bearing capacities in the range of that of natural cartilage are hence necessary [5].

Native articular cartilage exhibits a columnar orientation of cells with an anisotropic direction of the collagen fibers, which run vertically from the calcified cartilage towards the surface [11]. This alignment of the collagen fiber network is believed to play an important role in biomechanical functioning and the diffusional transport of water and macromolecules in the cartilage [12]. The design of an organized scaffold with a vertical orientation of microtubules to mimic native articular cartilage is hence an attractive strategy [13].

**Fig 1:**
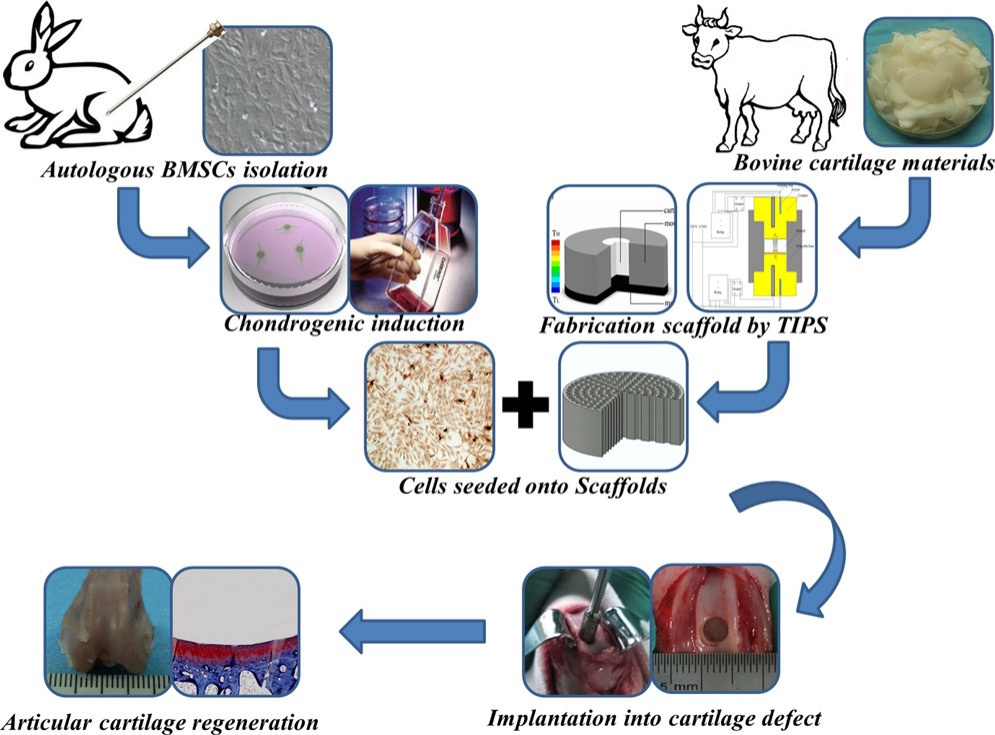
A schematic illustration of the experimental design for articular cartilage repair.

Previously, we have demonstrated that use of an oriented cartilage extracellular matrix (ECM)-derived scaffold in combination with chondrogenic bone mesenchymal stem cells (BMSCs) yields superior, mechanically functional TE cartilage within 4 weeks of implantation at subcutaneous sites in nude mice [14]. However, the therapeutic effects of oriented scaffold in situ in an articular cartilage defect remains to be explored. Thus, the purpose of the present study was to further assess the capacity of this organized oriented scaffold for repair of full-thickness cartilage defects in rabbits, over a 24-week post-operative period (Fig 1).

## Materials and Methods

### Animals and reagents

All animal experimental procedures used in this study were approved by the Institutional Animal Review Committee of Shannxi Hospital of Armed Police Force (Xi’an, China) (Permit Number: 2012-S102). All tissue culture reagents were from Sigma-Aldrich (St. Louis, MO, USA) unless specifically indicated.

### Fabrication and characterization of oriented scaffolds

**Fig 2:**
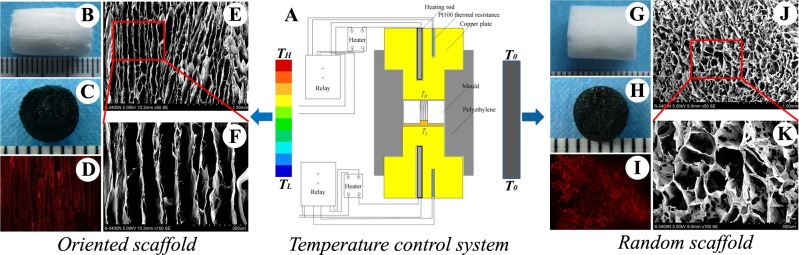
Overview of scaffold fabrication and characterization. Schematic of the temperature control system used to fabricate the oriented scaffold (A); macroscopic views of the oriented (B) and random scaffolds (G), oriented (C) and random (H) scaffolds stained dark-blue after cross-linking with genipin; SEM micrographs of the microtubules in the oriented scaffold (E, F) and the sponge-like pores in the random scaffold (J, K); Scaffold auto-fluorescence (red) after being cross-linked with genipin (D, I). T_H,_ high temperature; T_L,_ low temperature; T_0,_ no difference in temperatures between the top and bottom of the mould.

Oriented scaffolds were prepared using the previously described temperature gradient-guided thermal-induced phase separation (TIPS) technique [15]. Briefly, bovine cartilage slices were sectioned from femoral condyles (Laboratory Animal Center, Fourth Military Medical University, Xi'an, China), and re-formed to give ECM powder. Next, a 3% (w/v) suspension of the ECM powder in deionized water was infused into a cylindrical mould (diameter: 8 mm; height: 15 mm), which was then immersed in liquid nitrogen. This TIPS procedure was performed using a temperature control system designed by our group (Fig 2A). The system, which comprised a heating rod, Pt100 thermal resistance, a temperature controller, and a copper plate, provided a unidirectional stable temperature gradient (-80°C [T_L_] at the bottom of the molud to 0°C [T_H_] at the top of the mould) that allowed phase separation of the ECM solution and crystallization of the solvent. Next, the frozen samples were lyophilized in a freeze-dryer (Alpha 2–4, Chaist, Germany) for 24 h to produce longitudinally oriented microtubules. The scaffolds were then removed from the mould and cut into cylinders of 4 mm diameter and 4 mm thickness. The random scaffold was fabricated by traditional simple freeze-drying [16]. To improve the mechanical properties of both scaffolds, the samples were cross-linked with a 0.5% (w/v) solution of genipin (Wako Pure Chemical Industries, Osaka, Japan) for 48 h at room temperature. All scaffolds were sterilized by exposure to 20 kGy^60^Co radiation prior to cell culture and animal studies, and their morphology was observed by scanning electron microscopy (SEM; S-3400N, Hitachi, Tokyo, Japan). The porosity and density of the two types of scaffolds were analyzed according to previously reported methods [17], and their biomechanical properties were assessed by measuring the compressive modulus, as previously described [18].

### BMSCs isolation, chondrogenic induction, and seeding on scaffolds

Autologous BMSCs were obtained from the tibia of New Zealand White rabbits by bone marrow aspiration, as previously described [19]. Briefly, rabbits were anesthetized by injection of ketamine hydrochloride into the peritoneal cavity. Bone marrow was aspirated from the tibia using a 10 ml syringe containing 0.1 ml heparin (3000 U/ml saline solution), with a 16-gauge needle. Then the mononuclear cells were separated by centrifugation, and suspended in DMEM containing 20% FBS. Cells were cultured to 80% confluence, detached using 0.05% trypsin, and then subcultured in a chondrogenic induction medium containing high glucose DMEM, 10% FBS, 10 ng/mL TGF-β_3_ (PeproTech, Rocky Hill, NJ, USA), 1% ITS^+^ premix (BD, Franklin Lakes,NJ, USA), 10^−7^ M dexamethasone, 50 μg/mL ascorbic acid, 1 mM sodium pyruvate, 4 mM proline, and 1% antibiotics (penicillin and streptomycin) [16]. Then the cell density of the chondrogenic-induced culture was adjusted to 1×10^7^ cells/mL, and 50-μL cell suspensions were seeded onto each scaffold (5×10^5^cells/scaffold). The morphology and distribution of the cells within the scaffolds was observed by SEM. After bone marrow aspiration, the experimental rabbits were returned to their individual cages and allowed to move freely. A post-surgical antibiotic (gentamicin) was administered intramuscularly at 400,000 U per day for 3 days.

### Cell viability and proliferation within scaffolds

Cell viability in the cell-scaffold construct was evaluated at day 7 using a Live/Dead assay kit (Invitrogen, Camarillo, CA, USA), according to the manufacturer’s instructions [20]. Additionally, cell nuclei were stained with DAPI to improve cell visualization on the substrate [15], and cell proliferation within the scaffold was evaluated using the Cell Counting KIT-8 (CCK-8; Dojindo, Kumamoto, Japan) in accordance with the manufacturer’s protocol [20].

### Experimental design and surgical procedures

The autologous BMSC-scaffold constructs were implanted into the articular cartilage defects of New Zealand White rabbits. Briefly, a total of 27 3-month-old rabbits weighing between 2.5~3.0 kg were anesthetized by injection of ketamine hydrochloride into the peritoneal cavity. The knee joint was exposed through a medial parapatellar approach, and the patella was dislocated laterally. With the knees flexed, a hollow trephine was used to create a full-thickness articular cartilage defect in the femur trochlea, down to the subchondral bone (4 mm in diameter and 4 mm in depth). Then, the constructs was carefully inserted into the defect ensuring an adequate press-fit fixation while the scaffold was flush with the articular surface. The defect in the left joint was implanted with a cell-oriented scaffold construct (group I, 18 joints), and that in the right joint was treated with a cell-random scaffold composite (group II, 18 joints), while in the negative control group (group III, 9 rabbits, 18 joints) the defect was left untreated. No external fixation was performed after surgery, and the animals were allowed to move freely with total weight-loading. Animals were sacrificed by an intravenous overdose of pentobarbital at 6, 12, or 24 weeks post-operation, and the knee joints were harvested.

### Micro-CT scanning of the regenerated cartilage

For morphological observation, the specimens were investigated using desktop microcomputer tomography (Micro-CT; GE healthcare, Madison, USA). A 360-degree scan was carried out at a voltage of 80 kV, a current of 80 μA, and an exposure time of 2960 ms [3]. From the CT data set, a cylindrical region of interest (ROI) corresponding to the original defect location was selected for analysis [21]. Sliced CT data were three-dimensionally reconstructed.

### Histological examination and immunohistochemical analysis

The samples were fixed in 4% paraformaldehyde for 24 h and then decalcified in 10% EDTA for 4 weeks at room temperature. Specimens were dehydrated through increasing concentrations of ethanol, followed by paraffin embedding. All samples were sectioned at a thickness of 5-μm and stained with hematoxylin-eosin (HE), toluidine blue, and safranin O, according to standard protocols [16]. Semi-quantitative histomorphological analysis was carried out by two blinded observers to evaluate cartilage regeneration using the modified O’Driscoll grading scale, which apportions 0–26 points based on 10 individual parameters [22]. Collagen type II was detected immunohistochemically, using monoclonal anti-collagen type II (Invitrogen) antibodies, as previously described [19].

### Biomechanical analysis of the regenerated cartilage

For biomechanical and biochemical analyses, samples were first trimmed along the rim of the regenerated tissue using a trephine. Then, as per the method described by Hoenig et al. [9], samples were transferred to a stainless steel dish containing PBS at room temperature for the unconfined compression test (UCC), which was conducted using a universal material testing machine (Shimadzu, Kyoto, Japan). The height of each specimen was determined at a compressive force threshold of 0.05N. Five progressive strain loadings at 4% of the original cartilage height were performed with a test velocity of 0.01 mm/s. After each loading cycle, samples were subjected to a 2000-s relaxation phase to attain equilibrium. The Young's modulus value was then determined using the load and displacement data obtained at the end of each relaxation phase [14].

### Biochemical analysis of the regenerated cartilage

After biomechanical analysis, the DNA levels in the samples were quantified using the Quant-iT™ PicoGreen® dsDNA Assay Kit (Molecular Probes, Eugene, OR, USA), as previously described [14]. The total glycosaminoglycan (GAG) content was determined using a Blyscan™ Sulfated Glycosaminoglycan Assay kit (Biocolor, Carrickfergus, Northern Ireland, UK), based on the absorbance at 656 nm [14]. Total collagen was evaluated by the Sircol™ Soluble Collagen Assay kit (Biocolor), based on the absorbance at 555 nm [14].

### Statistical analysis

Statistical analysis was performed using the SPSS 15.0 software package. All values were expressed as the mean ± standard deviation. Data from the CCK-8 assay were analyzed using independent t-tests. The differences in biochemical content and Young’s modulus values between groups were analyzed by one-way ANOVA. Differences where *p* <0.05 were considered statistically significant.

## Results

### Characteristics of oriented scaffolds

Fabrication and characterization of the oriented and random scaffolds are shown in Fig 2. Following cross-linking with genipin, the scaffold color changed from white to dark blue (Fig 2B, 2C, 2G and 2H). SEM analysis revealed that the pores within the oriented scaffolds were microtubule-like and arranged in parallel in the vertical plane, and that the oriented microtubules (diameter, 95.2 ± 20.9μm) were interconnected (Fig 2E and 2F). In sharp contrast, the pores within the random scaffolds were distributed randomly and uniformly, with a macropore diameter of 97.3 ± 27.5 μm (Fig 2J and 2K). Interestingly, the ECM-derived scaffold exhibited autofluorescence (red) following genipin crosslinking (Fig 2F and 2I). There was no significant difference in porosity (91.6% ± 3.1% and 92.2% ± 3.0%, respectively) or density (72.6 ± 6.8 μg/mm3 and 69.1 ± 7.4 μg/mm3, respectively) when comparing the oriented and random scaffolds. However, the Young's modulus value for the oriented scaffold was 3-fold higher than that of the random scaffold (89.35 ± 7.96 kPa vs. 36.20 ± 4.67 kPa; p < 0.05).

### Cell distribution, viability, and proliferation within scaffolds

**Fig 3:**
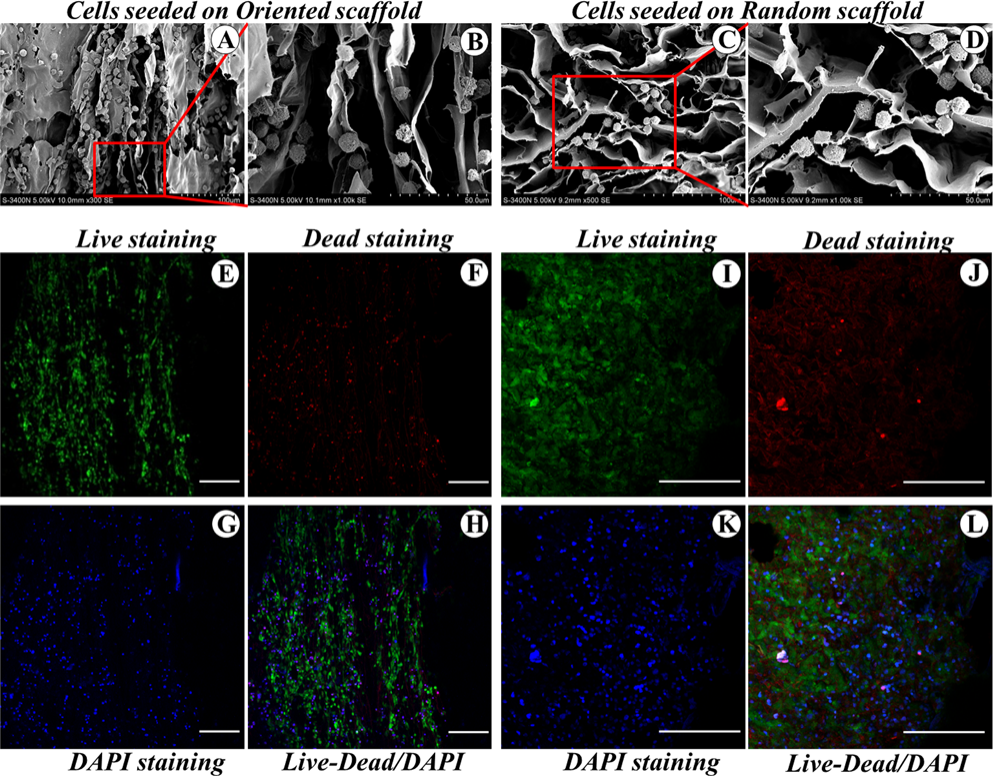
Cell distribution and viability within the scaffolds. Distribution and morphology of the differentiated BMSCs cultured on the oriented (A, B) and the random (C, D) scaffolds; Live/Dead staining of BMSCs within the oriented (E, F) and the random (I, J) scaffolds on day 7; Examination of distribution of cell nuclei (blue: DAPI-stained) (G, K) in oriented (H) and random scaffold (L).Scale bar: 200 μm.

Analysis of cell growth by SEM revealed that while the chondrogenic BMSCs were aligned along the microtubules in the oriented scaffold (Fig 3A and 3B), they were distributed homogeneously and randomly on the surface of the pore walls in the random scaffold (Fig 3C and 3D). There was no significant difference in the cell morphology when comparing BMSCs in two groups throughout the period of the experiment. Cell viability evaluation by Live/Dead staining followed by image analysis showed > 95% cell viability in two groups (live cells stained fluorescent green, while dead cells stained red; Fig 3E, 3F, 3I and 3J). Nucleus visualization using DAPI staining revealed that cells were aligned along the length of the channel within the oriented scaffold (Fig 3G). In contrast, cells within the random scaffold showed a random spatial orientation (Fig 3K)

**Fig 4:**
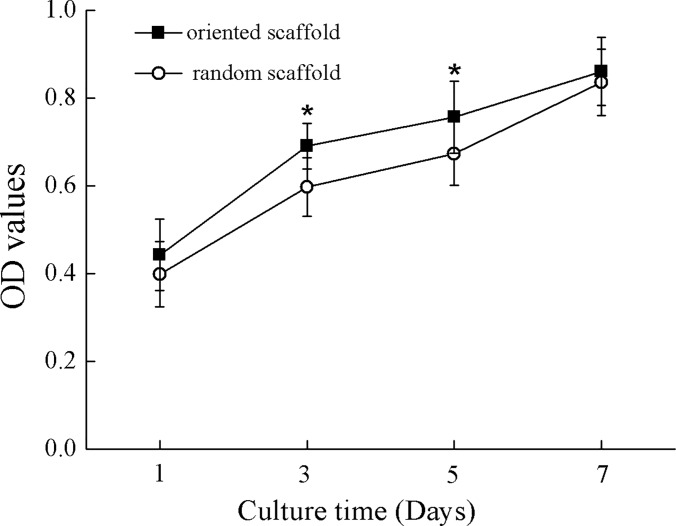
Cell proliferation assay within scaffolds. Proliferation of differentiated BMSCs seeded on the oriented scaffold (filled squares) or the random scaffold (circles). *, *p* < 0.05.

Cell proliferation within the scaffolds was analyzed by the CCK-8 test after culture for 1, 3, 5, or 7 days (Fig 4). The optical density (OD) values for the oriented scaffolds were higher than those for the random scaffolds at day 3 and 5 (*p* < 0.05), but this difference was not maintained when examined at day 7, possibly due to the increase in cell numbers and ECM accumulation within the scaffolds over time [23].

### Histological and immunohistochemical assessments of cartilage repair

**Fig 5:**
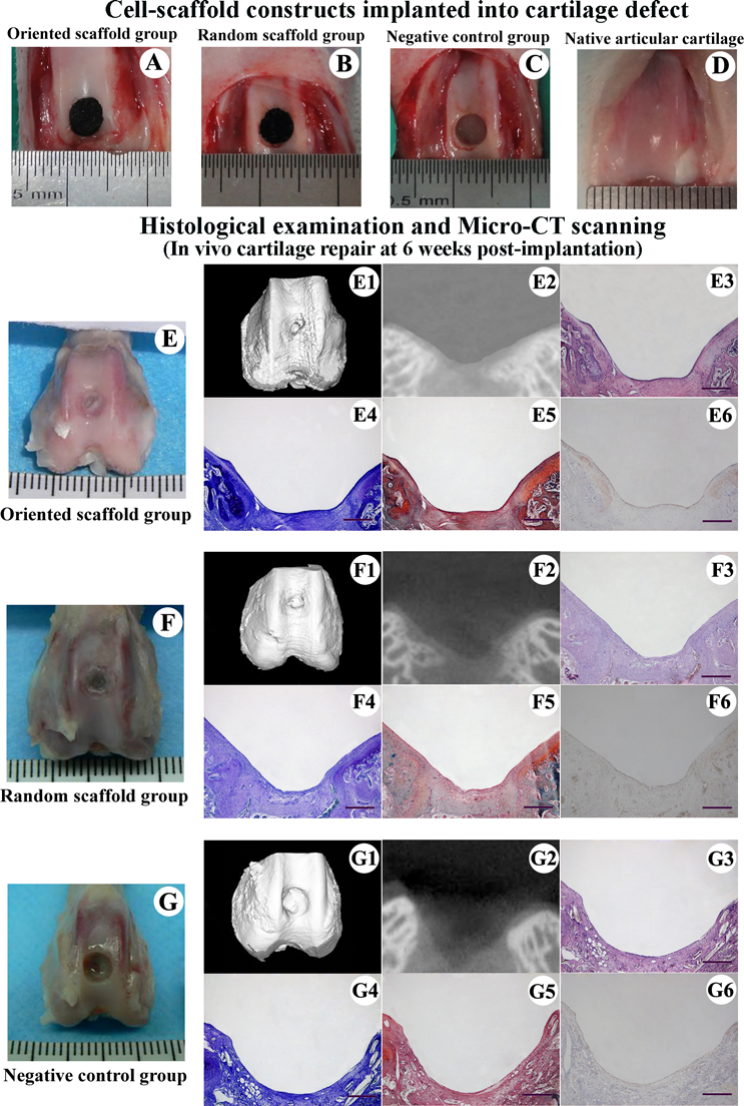
Experimental design and cartilage repair at 6 weeks post-implantation. Cell-scaffold constructs were implanted into the cartilage defect: oriented scaffold group (A), random scaffold group (B), and negative control group (C). The native articular cartilage (D) was utilized as normal control. Gross appearance of the knee joint (E, F, G) and micro-CT scanning (E1, E2, F1, F2, G1, G2) of the defect at 6 weeks. HE (E3, F3, G3), toluidine blue (E4, F4, G4), safranin O (E5, F5, G5) and collagen type II staining (E6, F6, G6) of the regenerated cartilage. Scale bar: 1 mm.

**Fig 6:**
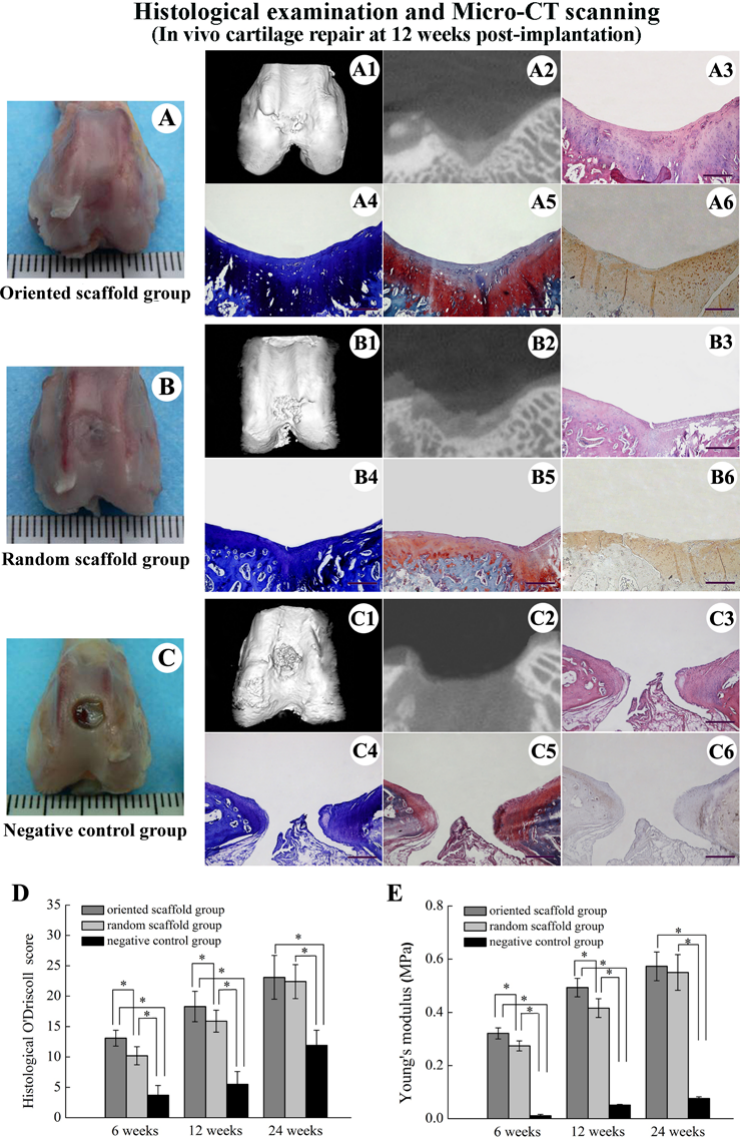
Articular cartilage repair at 12 weeks and evaluation of regenerated cartilage through histomorphological and biomechanical grading. Gross appearance of the knee joint (A, B, C) and micro-CT scanning (A1, A2, B1, B2, C1, C2) of the defect at 12 weeks. HE (A3, B3, C3), toluidine blue (A4, B4, C4), safranin O (A5, B5, C5) and collagen type II staining (A6, B6, C6) of the regenerated cartilage (scale bar: 1 mm). Histomorphological (O’Driscoll) scores (D) and Young's modulus values (E) of the regenerated cartilage in the two scaffold-implanted groups, throughout the experimental period. *, *p* < 0.05.

The articular cartilage defects of oriented scaffold group (Fig 5A), random scaffold group (Fig 5B), and negative control group (Fig 5C) were present in Fig 5. When examined 6 weeks after implantation, the defects still showed a rough and sunken surface in both the oriented and random scaffold groups, with the regenerated cartilage being thinner than the surrounding native cartilage and the interface between two being clearly identifiable (Fig 5E and 5F). Toluidine blue and safranin O staining revealed that while the cartilage defect in the oriented scaffold group had been repaired by a mixture of fibrocartilage and fibrous tissue (Fig 5E4 and 5E5), only fibrous tissue was observable in the random scaffold, with no cartilage formation apparent in the defect and GAG being present at low levels in the central area (Fig 5F4 and 5F5). Micro-CT scanning revealed incomplete filling and concave defects with some low-density tissue regeneration in both experimental groups (Fig 5E1, 5E2, 5F1 and 5F2). As expected, the defect in negative control group was not repaired, and a vacancy was observable in the center (Fig 5G1–5G6). The total histomorphological score was higher in the oriented scaffold group compared to the random scaffold group (13.12 ± 1.33 vs. 10.2 ± 1.51; *p* < 0.05), and both these scores were higher than that of the negative control group (3.73 ± 1.61; *p* < 0.05; Fig 6D).

When examined 12 weeks after transplantation, the defect in the cell-oriented scaffold group was almost smooth, with gradual replacement by a newly formed mixture of cartilage-like and fibrocartilage tissue, with more organized cell distribution and appearance of columnar structures (Fig 6A1–6A6). In contrast, the defect in the random scaffold group was almost covered by a rough cartilage-like tissue, the newly formed tissues were separated from the normal cartilage by a clearly distinguishable depression, and the cell organization was irregular with mixed clusters (Fig 6B1–6B6). Micro-CT scanning showed that both groups showed neo-tissue formations, including cartilage and subchondral bone at the peripheral areas, with these tissues developing toward the center of the defect in both groups (Fig 6A1 and 6B1). In contrast, the defects in the negative control group showed only partial repair, mainly at the area adjacent to the host tissue. This area showed disordered fibrous tissue, with a vacancy visible at the defect center (Fig 6C1–6C6). The histomorphological scores were 18.32 ± 2.54 in the oriented group, compared to 15.92 ± 1.89 in the random group (*p* < 0.05), and both scores were notably higher than that in the negative control group (5.53 ± 2.15, *p* < 0.05; Fig 6D).

**Fig 7:**
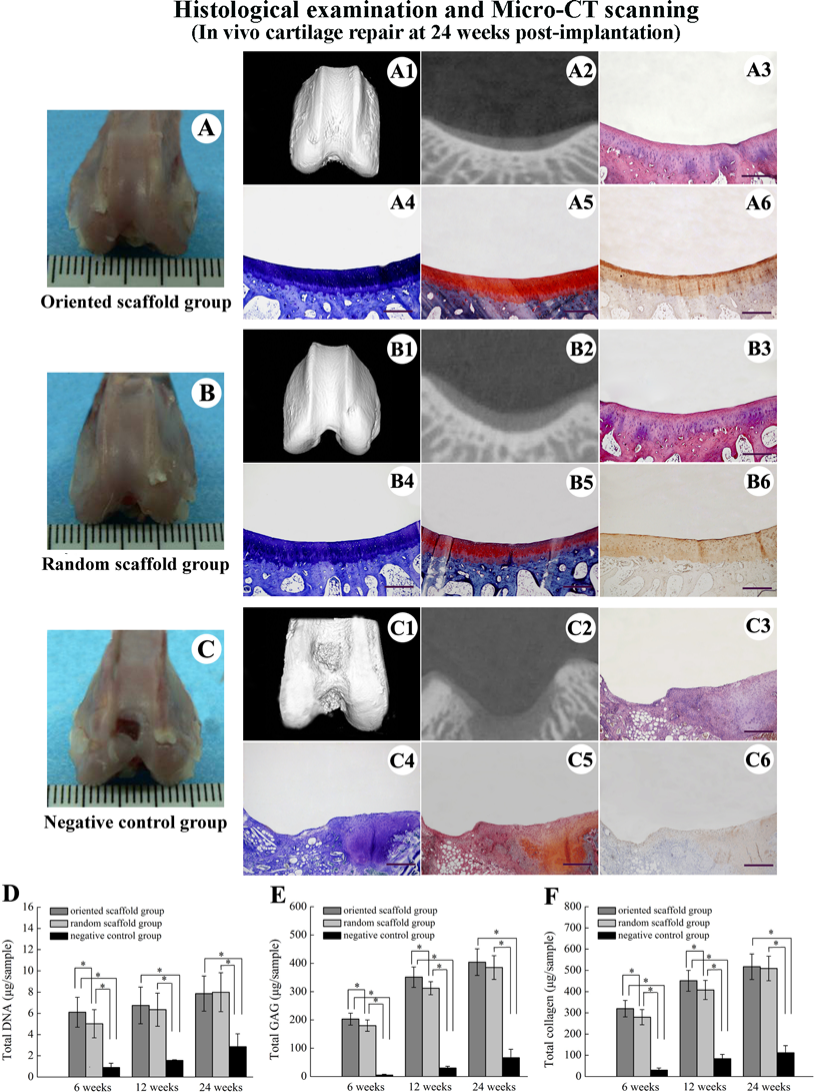
Articular cartilage repair at 24 weeks and biochemical evaluation of regenerated cartilage. Gross appearance of the knee joint (A, B, C) and micro-CT scanning (A1, A2, B1, B2, C1, C2) of the defect at 24weeks. HE (A3, B3, C3), toluidine blue (A4, B4, C4), safranin O (A5, B5, C5) and collagen type II staining (A6, B6, C6) of the regenerated cartilage (scale bar: 1 mm). Biochemical evaluation showing total DNA (D), total GAG (E) and total collagen (F) in the regenerated cartilage in the two experimental groups. *, *p* < 0.05.

When examined 24 weeks after surgery, the surface of the regenerated defect in the oriented scaffold group was generally smooth and flat, with the interface between the neo-cartilage and surrounding normal tissue being nearly indistinguishable (Fig 7A). Toluidine blue, safranin O and immunohistochemical staining indicated that the original defect had been filled in by hyaline cartilage-like neo-tissue, and that the cells were organized in columns and clusters that were aligned vertical to the articular surface (Fig 7A3–7A6). At this time point, the defect surfaces in the random cartilage group showed thicker, hyaline cartilage-like tissue that appeared to be well integrated with the surrounding normal cartilage, thus displaying good repair (Fig 7B). The cell morphology and distributions in the neo-cartilage were almost identical to that of the host natural cartilage (Fig 7B3–7B6). Furthermore, the neo-cartilage showed strong staining for collagen type II in both the experimental groups (Fig 7A6 and 7B6), and micro-CT scanning revealed complete regeneration of the cartilage and subchondral bone in both groups (Fig 7A2 and 7B2). In contrast, the defect in negative control group was filled with only fibrous tissue (Fig 7C1–7C6). The histomorphological scores for the oriented and random scaffold groups were similar (23.1 ± 3.6 and 22.4 ± 2.91, respectively), and both were markedly higher than that of the control group (11.90 ± 2.53, *p* < 0.05; Fig 6D).

### Biomechanical evaluation of the regenerated cartilage

The Young's modulus values for the regenerated cartilage in the two operated groups were significantly higher than that in the control group, at all the time points examined. When comparing the two operated groups, Young’s modulus values were significantly higher in the oriented scaffold group compared to the random group at the early time points (6 and 12 weeks; *p* < 0.05), but this difference was abrogated by 24 weeks (Fig 6F). Importantly, the compressive modulus of the newly formed cartilage in the oriented scaffold group (0.573 ± 0.055 MPa) appeared to approach that of native cartilage (0.636 ± 0.051 MPa) at 24 weeks post-implantation. Thus, based on a biomechanical evaluation, use of the oriented scaffold promoted well-organized cartilage repair, producing neo-cartilage with a mechanical strength similar to that of normal cartilage.

### Biochemical evaluation of regenerated cartilage

We also examined the levels of DNA, GAG, and collagen in the samples from both the oriented and random scaffold groups; the amounts of all three increased with an increase in the post-implantation time, and were significantly higher (*p* < 0.05) than the corresponding values in the negative control group throughout the experimental period (Fig 7D, 7E and 7F). The DNA content of the oriented scaffold group samples was higher than that of the random group (*p* < 0.05) at 6 weeks, but this difference was abrogated at 12 weeks and 24 weeks (7.86 ± 1.65μg/sample vs. 7.99 ± 1.83 μg/sample; Fig 7D). GAG deposition in the orientated scaffold group samples was higher than that in the random group (*p* < 0.05) at 6 and 12 weeks, but appeared to be similar by 24 weeks (404 ± 47 μg/sample vs. 385 ± 35 μg/sample; Fig 7E). Similarly, collagen production in the oriented scaffold group samples was higher than that in the random group samples at 6 and 12 weeks (*p* < 0.05), but not at 24 weeks, where the levels were similar (517 ± 63 μg/sample vs. 509 ± 54 μg/sample; Fig 7F). By the end of the observation period (24 weeks), the total DNA, GAG, and collagen levels in both the experimental groups were similar to that of normal cartilage (7.78 ± 0.35μg/sample, 460 ± 30 μg/sample and 535 ± 45 μg/sample, respectively).

## Discussion

In this study, we examine the importance of scaffold orientation on the in situ repair of articular defects in a rabbit model. We synthesized a novel oriented scaffold by TIPS technology, and tested the efficacy in this scaffold in combination with chondrogenic BMSCs in articular cartilage regeneration, and showed enhancement in the repair of full-thickness articular cartilage defects with improvement in the biomechanical properties of the newly generated cartilage.

While cartilage repair approaches are required to restore not only the structure, but also the biomechanical functioning of articular cartilage, existing TE technologies fall short on the latter [15]. In this study, implantation of an oriented scaffold-BMSC construct produced neo-cartilage with enhanced biomechanical properties and accelerated improvement of parameters such as the Young's modulus observable at the early time points. In designing an oriented scaffold, we took into consideration that its bionic structure may play a key role in determining the biomechanical characteristics of the neo-cartilage. In recent years, diverse orientation-structured scaffolds have been producing from various materials [24], using varied techniques [25]. The studies examining these have uniformly reported that such structural orientation produced scaffolds with higher strength and an increase in the compression modulus in vitro [17, 20]. The novel oriented scaffold used in this study has been described in an earlier study from our group [14], and as previously reported, this scaffold showed Young's modulus values higher than that of porous random scaffolds, in the dry state in vitro [18]. This improvement in biomechanical characteristics may be attributable to the increase in the thickness of the microtubule walls, which confers an increased capacity to withstand compressive stress [20]. Thus, while the effects of an oriented structure on the biomechanical properties have been well examined in vitro, this topic has not been examined in vivo. Therefore, to test the effect of scaffold orientation on in situ cartilage regeneration, we cultured the oriented scaffold with chondrogenic BMSCs and implanted these constructs into full-thickness articular cartilage defects in rabbits, and evaluated the mechanical strength in vivo.

Histological and immunohistochemical observation of the in vivo implanted scaffolds showed that the oriented microtubule could induced cells migrate into the core of scaffold; this structure may result in an enhancement of mechanical properties, to protect cells from early critical compression before sufficient ECM was deposited [26]. In early post-implantational period, Young's modulus values for the regenerated cartilage were higher in the oriented scaffold group compared to the random scaffold group (*p* < 0.05). This may be attributable to the three-dimensional environment of the oriented scaffolds, which could affect the direction of extracellular matrix deposition, as cells migrating into the scaffold would align with the microtubules and follow the channels arranged by the scaffold [20]. Consequently, regular cell organization with columnar clusters would result, along with vertical alignment of the gradually formed giant collagen bundles in the neo-cartilage, providing the joint with powerful support for compression in the vertical direction.

In contrast, efficient cell adhesion and proliferation were observed for both the oriented and random ECM-derived scaffolds in vitro, which may be attributable to the native components of these scaffolds [7]. Previous studies have demonstrated that the ECM-derived scaffold, which retains much of the cartilage GAG and collagen type II [27], provides a natural microenvironment capable of supporting BMSC attachment, proliferation, and differentiation into chondrocytes [28]. Through the CCK-8 assay, we verified that differentiated BMSCs seeded on both types of scaffolds underwent rapid growth in the first week of culture, confirming previously reported trends describing the proliferation of chondrocytes on porous ECM scaffolds [16]. Moreover, cell proliferation on the oriented scaffold was higher than that on the random scaffold at days 3 and 5 of culture; this increase may be attributed to the oriented, congruently aligned and interconnected structure of the microtubules, which facilitates the transport of nutrients and the exchange of metabolites both in vitro and in vivo. However, this advantage was reduced by day 7, due to the increase in cell numbers and ECM accumulation. Meanwhile, the SEM and fluorescent microscopy data indicated that the oriented scaffold serves as a guide for BMSC attachment and alignment along the vertically oriented microtubules, thus mimicking the physiological structure of native cartilage. These results demonstrate that the ECM-derived scaffold with a biomimetic oriented structure and biochemical composition not only enhances cell adherence and proliferation, but also induces cell migration into the scaffold along the vertically oriented microtubules, thus promoting ECM deposition.

Histological and immunohistochemical analysis revealed efficient cartilaginous matrix secretion and articular cartilage regeneration in both the oriented and random scaffold groups in vivo. More importantly, knee joints implanted with the oriented construct showed superior cartilage formation and bridging of the patellar groove defect, due to enhanced cartilage repair early after implantation. These results may be attributable to the vertically aligned microtubules in the oriented scaffold, which may facilitate the migration of cells into the center of defect and result in neo-cartilage growth from the bottom. Biochemical analyses showed an increase in total GAG and collagen levels in the post-implantation time in both groups, consistent with previous studies [29]. Nonetheless, GAG and collagen deposition in the oriented scaffold group occurred more rapidly, with higher levels compared to that in the random group at 6 and 12 weeks post-operation. This may also be attributable to the increased cell migration to the central of cartilage defect in the oriented scaffold, with resultant abundant secretion of cartilage ECM components such as GAG and collagen type II. Additionally, the oriented structure may also support improved transport of nutrient molecules and the exchange of metabolites during the early stages of repair.

According to a previous study, cartilage can be regarded as a biphasic material with complex mechanical properties such as anisotropy, nonlinearity, and viscoelasticity [30]. In natural articular cartilage, stiff and elastic collagen fibers allow the cartilage to resist lateral expansion on axial compression by maintaining a compact framework [31]. GAG or aggrecan, which are tightly immobilized onto a collagen network, can retain a very high number of water molecules. These highly concentrated and hydrophilic proteoglycans provide a swelling pressure within the ECM, which is constrained by a tight collagen network to resist external force [32]. Upon greatly increased pressure, a little water may be squeezed out, resulting in reversible deformation of cartilage and a temporary increase in the area of contact. Meanwhile, majority of water molecules are held in place by GAG and remain condensed at their original location, thus contributing to the compression stiffness of cartilage [33]. GAG, collagen, as well as their interaction provide articular cartilage with a unique ability to undergo reversible compression and strength to withstand mechanical stress [34]. Thus, to a certain extent, enhanced GAG and collagen deposition plays an important role in enhancing the biomechanical properties of the regenerated cartilage. This may also explain why the Young's modulus values were higher in the oriented scaffold group during the early phase of transplantation.

In present study, a rabbit model was used to assess cartilage regeneration and function following implantation of a novel oriented scaffold-BMSC construct into a full-thickness cartilage defect, with promising results. This oriented scaffold should be further investigated in a large animal model such as sheep, with assessment of knee joint repair and function over a long (1 year) period. Additionally, instead of measures such as GAG and collagen deposition to indirectly evaluate BMSC differentiation and proliferation within scaffolds, more accurate methodology such as quantitative polymerase chain reaction (PCR) should be applied, to further evaluate the proliferation and specificity of differentiation.

## Conclusion

In this study, we tested the cartilage repair possibility of a novel oriented cartilage ECM-derived scaffold produced using TIPS technology in a rabbit model, and showed that this scaffold, whose compressive modulus was superior to that of a random scaffold, in combination with differentiated BMSCs successfully repaired full thickness cartilage defects with a greatly improved Young's modulus within a 24-week period. Moreover, use of this oriented scaffold significantly enhanced biomechanical properties of the regenerated cartilage by providing a longitudinal oriented structure, thus indicating the potential application of this strategy in the clinic.
